# Translation, cultural adaptation and validation of the Tamil version of the Cardiff Acne Disability Index (CADI) in Sri Lanka

**DOI:** 10.1186/s41687-024-00782-0

**Published:** 2024-09-26

**Authors:** Shamini Prathapan, Achala Liyanage, Sailakshmi Logeeswaran, Wathsala Ratnayake, Lilangi Devapriya, Jennifer Perera

**Affiliations:** 1 Department of Community Medicine, University of Sri Jayewardanapura, Nugegoda, Sri Lanka; 2 Department of Community Medicine, Faculty of Medicine, University of Ruhuna, Karapitiya, Sri Lanka; 3 Faculty of Medicine, University of Colombo, Colombo, Sri Lanka

**Keywords:** Acne vulgaris, Cardiff Acne Disability Index-Tamil, Validation, Quality of life

## Abstract

**Background:**

Assessment of QoL has become an essential component in the holistic care of patients with acne. The Cardiff Acne Disability Index (CADI) is used globally to assess quality of life (QoL) in patients with acne. This study was done to validate CADI in Tamil, as 90 million of the global population are native speakers and Tamil is an official language of several countries.

**Methods:**

CADI was translated and validated into Tamil according to published guidelines. The Tamil versions of both CADI and Dermatology Life Quality Index (DLQI), was administered to 150 Sri Lankan young adults with acne. The clinical severity was assessed using the Global Acne Grading System (GAGS). Discriminant validity was tested by comparing the results of CADI with those of GAGS and DLQI, using reliability, validity, Cronbach’s alpha, and Spearman’s correlation coefficient measurements. Construct validity was assessed by factor analysis.

**Results:**

70% were female, and the mean age was 25.1 (SD, 5.2). The majority (91.3%) had acne of mild to moderate severity when measured by GAGS. CADI-Tamil showed high internal consistency and reliability (Cronbach’s alpha coefficient = 0.83). The CADI total score showed a strong correlation (0.86) with that of DLQI. The correlation between CADI and GAGS was low, whereas CADI had a high and significant correlations with the DLQI. The construct validity explained 61% of the variability.

**Conclusions:**

The CADI-Tamil is a reliable and valid tool for assessing the QoL of Tamil speaking patients with acne. This tool will help clinicians understand the patient’s perspective on acne.

**Supplementary Information:**

The online version contains supplementary material available at 10.1186/s41687-024-00782-0.

## Introduction

Acne is an inflammatory disorder of the skin and a common dermatological condition typically associated with the adolescent population, which sometimes persists into adulthood. Nearly two thirds of adults in the age group of 20 to 29 years and approximately 43% of the 30–39-year-olds experience acne [1]. Acne is characterised by sebum overproduction, follicular hyperkeratinisation, and increased release of inflammatory mediators. Androgens, microbes, and other pathogenetic influences are trigger factors for the development of acne [2–4]. This can lead to a high disease burden, especially when it is associated with impaired health-related quality of life (QoL), which has been considered critical for overall clinical evaluation [5].

Acne being a common disease among dermatology patients, various tools have been developed and validated to measure the QoL of such patients. The importance of measuring QoL begins with the initial evaluation of patients in order to understand and measure the progress of treatment from a clinical point of view and to compare the outcomes of clinical studies from a research point of view. The commonly used patient-reported QoL outcome measures are the Cardiff Acne Disability Index (CADI), specific for acne, [6, 7] and the Dermatology Life Quality Index (DLQI) [8], a generic tool used for assessing the QoL of patients affected by any type of skin disease. The global acne grading system (GAGS) is the most common tool that assesses clinical severity [9].

CADI consists of five short questions related to distinctive situations in daily life and is a useful tool to measure acne-specific QoL in busy clinic settings. The total score has a maximum of 15 and a minimum of 0, with higher scores indicating a more impaired quality of life [7]. CADI was first developed for the English population, and has been translated to and validated in many other languages subsequently. Currently, there is no validated CADI in the Tamil Language, which has its roots in the Dravidian language family. There are close to 90 million native Tamil speakers worldwide, and they are distributed predominantly in Asia. The countries in Asia that have a significant percentage of native Tamil speakers in their respective populations include, Sri Lanka (18%), Southern India (5.8%), Singapore (4.4%), Malaysia (3.9%), Myanmar (2.1%), and Mauritius (0.8%) [10]. Tamil is an official language both in Singapore and Sri Lanka. Hence, the present study was carried out for the translation of the CADI into Tamil and then to validate the construct and assess reliability among Tamil-speaking adults in Sri Lanka as a patient-reported outcome measure tool.

## Methods

### Translation

After obtaining permission from the original author, the English questionnaire was translated independently to Tamil by two bilingual medical professionals well versed in both Tamil and English as per widely recommended forward-backward translation methods [11]. Their first language was Tamil. The translations carried out by a person in his or her first language are more likely to accurately reflect the nuances of that language [12].

During the translation process, the translators were asked to pay special attention to ensuring conceptual and normative equivalence, rather than focusing merely on preserving the literal meaning of each word. Technical terms and long sentences were avoided to enhance the understanding of the target groups. Any discrepancies and ambiguities between the translated versions and the original English version were resolved by consensus between the bi lingual translators, and a finalised Tamil-translated version was agreed upon for backward translation. Two independent native English speakers, who were completely blind to the original English version, translated the Tamil version back into English. Henceforth, the two independent translators, together with the team of translators, compared the English translation with the original tool to determine any discrepancies or conceptual errors. This translated tool was sent to a team of experts to assess its validity. The appropriateness of the wording and acceptability in the local context were looked into. For the cognitive debriefing, ten Tamil-speaking, Sri Lankan university students suffering from acne were selected to check the face validity of the finalised questionnaire with respect to comprehensibility, ambiguity of the items, and relevance to the social context. The final, refined version was accepted by the original authors of CADI for validation.

### Validation

The final version of the translated questionnaire was tested for its reliability and validity among study participants with different grades of severity of acne from both Universities of Colombo and Sri Jayewardanapura, Sri Lanka. One hundred and eleven university students and 39 employees, who are native Tamils and having acne of any degree of severity, were selected as study participants. The validation study was conducted from April to June 2023. Details about the study were included in an advertisement and displayed in the public areas of the university in both Tamil and English. The volunteers who were interested in participating in the study were requested to speak to the chief investigator to obtain details of the study. The eligible volunteers were given a date to participate in the study. After obtaining written informed consent from eligible participants, basic demographic data were collected using the case record form. They were asked to complete the Tamil CADI questionnaire and were asked to record the time taken to complete CADI and comment on its clarity and wording on a scale of 1–4 as poor, average, good, and very good.

The participants were also evaluated for the severity of acne using the Global Acne Grading System (GAGS) [9] by two medical graduates after being trained by a specialist dermatologist. The GAGS considers six locations, including five regions in the face and one in the trunk. For each region, one factor is assigned. Each type of acne lesion is given a value depending on its severity (0 = no lesion; 1 = comedones; 2 = papules; 3 = pustules; 4 = nodules). The score for each area is calculated by the formula: local score = factor × grade (0–4). The global score is the sum of local scores, which will determine the severity of acne: a score of 1–18 is considered mild; 19–30 is moderate; 31–38 is severe; and > 39 is very severe. This system has been proven to be accurate, showed minimal variability in intra-observer and inter-observer gradings, and has been widely used in research involving acne grading. The DLQI is a ten-item questionnaire that assesses the effect of acne in general on QoL. The total score ranges from 0 (no impact of skin disease on QoL) to 30 (maximum implications for QoL). The DLQI total score is interpreted as: 0–1 having no effect on the patient’s life, 2–5 having a small effect, 6–10 having a moderate effect, 11–20 having a very large effect, and 21–30 having an extremely large impact on the patient’s QoL [8].

The comparison of the CADI scores with the DLQI and GAGS scores was used to assess the discriminant validity. The statistical significance of the observed differences was set at 0.05. The reliability of the CADI was assessed by internal consistency, which is how many items within a domain are correlated to give the same result. This was assessed by calculating the Cronbach’s alpha coefficient. Estimates of a magnitude of 0.70 or greater were considered satisfactory. A correlation coefficient (Cohen’s Kappa) of 0.60 or above was considered a good level of agreement between scores. The statistical analysis of the data was done using SPSS version 22 (SPSS Inc., Chicago, Illinois, USA).

## Results

Results Among the 150 participants, the mean age of the study participants was 23.14 years, with a standard deviation (SD) of 5.3. Around 67% of the study participants were between the ages of 21 and 25. Nearly 70% were female, with almost 83% being of Tamil ethnicity, practicing the Hindu religion, and not married (87%). Two-thirds of the participants had a degree or were on their way to obtaining a degree. Three-fourths were obtaining a monthly income of around US$33 (Table 1).

**Table 1:** Sociodemographic characteristics of the study population

	Number/ Mean (SD) (*n* = 150)	Percentage
Age (mean/SD)	25.14 (5.2)	
Age		
< 20 years	11	7.3
21–25 years	100	66.7
26 years and more	39	26.0
Sex		
Male	45	30.0
Female	105	70.0
Ethnicity		
Tamil	124	82.7
Muslim	26	17.3
Religion		
Hindu	111	74.0
Christian/Catholics	13	8.7
Islam	26	17.3
Marital status		
Married	23	15.3
Others	127	84.7
Education		
Up to advanced level	38	25.3
Diploma/Vocational Training	17	11.3
Degree	91	60.7
Postgraduate qualification	4	2.7
Income		
Less than Rs 10,000 (US$ 33)	107	71.3
Rs 10,000–25,000 (US$ 33–84)	10	6.7
Rs. 25,001–50,000 (US$ 84–168)	27	18.0
Rs.50,001–75,000 (US$ 169–252)	5	3.3
More than Rs. 75,001 (US$ 252–336)	1	0.7

In order to establish discriminant validity, the DLQI and GAGS were compared with the CADI, as shown in Table 2.

**Table 2:** Comparison of CADI grading with DLQI and GAGS severity

	CADI				χ2; df; *p* value
	No impairment (*n* = 32) (%)	Mild impairment (*n* = 93) (%)	Moderate impairment (*n* = 20) (%)	Severe impairment (*n* = 5) (%)	
**DLQI**					
No effect at all	27 (84.4)	34 (36.6)	0 (0.0)	0 (0.0)	172.6; 9; 0.001
Small effect	5 (15.6)	50 (53.8)	3 (15.0)	0 (0.0)	
Moderate effect	0 (0.0)	8 (8.6)	10 (50.0)	0 (0.0)	
Very large effect	0 (0.0)	1 (1.1)	5 (25.0)	1 (20.0)	
Extremely large impact	0 (0.0)	0 (0.0)	2 (10.0)	4 (80.0)	
**GAGS severity**					
Mild	31 (96.9)	84 (90.3)	17 (85.0)	5 (100.0)	2.89; 3; 0.41
Moderate	1 (3.1)	9 (9.7)	3 (15.0)	0 (0.0)	
Severe	0 (0.0)	0 (0.0)	0 (0.0)	0 (0.0)	

Among those with no impairment in the CADI tool, 84% had no effect at all with the DLQI assessment, while 97% were found to have mild severity when assessed by the GAGS index. Similar observations were also seen at the other end of the grading, in which all 4 (80%) participants with severe impairment with the CADI tool were found to have an extremely large effect on the DLQI QoL tool. The statistical significance of the observed differences was assessed using the chi squared test, and there was a significant difference between the CADI and the DLQI (*p* = 0.001), whereas a statistically significant difference was not observed when compared to the GAGS severity scale, exposing the validity of the CADI and the DLQI on a categorical basis.

The mean Tamil CADI score obtained in the study was 3.03 with a SD of 3.2, which indicates a slight impairment in the quality of life of the population studied. As shown in Table 2, discriminant validity was assessed by comparing the CADI scores with the DLQI and GAGS severity scores. The CADI item correlations were stronger with DLQI when compared to GAGS. Except for the 5th item (*r* = 0.58) in CADI, all the other items showed a moderate or strong correlation with DLQI. However, the CADI items showed a poor correlation with GAGS (Table 3).

**Table 3:** Spearman’s rho correlation between GAGS and DLQI with CADI

CADI	GAGS (*p*)	DLQI (*p*)
CADI Total score	0.287 (0.001)	0.861 (0.001)
CADI Q1	0.265 (0.001)	0.762 (0.001)
CADI Q2	0.196 (0.02)	0.724 (0.001)
CADI Q3	0.114 (0.165)	0.668 (0.001)
CADI Q4	0.206 (0.01)	0.639 (0.001)
CADI Q5	0.305 (0.001)	0.580 (0.001)

To test the construct validity, an exploratory factor analysis was adopted with principal component analysis with Varimax rotation. The first question on “being aggressive, frustrated, or embarrassed” loaded highest on the component. However, all items loaded highly on the component with the least correlation of 0.65. This analysis showed that there was only one factor explaining the scale, explaining 60.5% of the sample variability, which shows one dominant underlying mechanism (Table 4).

**Table 4:** Factor loadings for the five items of the Tamil-CADI and the variance

Item	Component 1
As a result of having acne, during the last month have you been aggressive, frustrated or embarrassed?	0.875
Do you think that having acne during the last month interfered with your daily social life, social events or intimate personal relationships?	0.836
During the last month have you avoided changing public facilities or wearing swimming costumes because of your acne?	0.652
How would you describe your feelings about the appearance of your skin over the last month?	0.798
Please indicate how bad you think your acne is now	0.704
% of variance / % cumulative	60.5%

The internal consistency assessed by Cronbach’s alpha was 0.83, indicating good consistency of the CADI-Tamil instrument. The time required for completion of the study was an average of 3 min.

## Discussion

This study aimed to translate and validate the Tamil version of the CADI instrument for quality-of-life assessment in acne patients. Validated translation methods, cultural adaptation techniques, and psychometric analysis, were followed in developing the Tamil CADI and there were no significant structural alterations in the items or in the responses in the Tamil version.

The study population comprised subjects with facial acne enrolled by a passive search among the university graduates, and the study population characteristics are similar to those of other studies that used this method to evaluate the impact of acne on QoL. These results are consistent with the findings of previous studies, where most of the patients with acne had mild impairment (*n* = 93; 62%) with only a minority (3.3%) having more severe levels of acne [13, 14].

The analysis shows that the Cronbach’s α coefficient values reflect a high level of internal consistency. The correlations of CADI scores between the GAGS and the DLQI reinforce the questionnaire’s unity with QoL and not with the severity of the disease, and they also maintain the CADI instrument’s multidimensional characteristics as a tool to assess QoL. Validation studies of QoL tools, including CADI, have been compared and correlated using DLQI. Among the 16 validation studies done previously on the CADI tool, 12 used DLQI as the comparator for assessing correlation, and all showed a good correlation with CADI, similar to the present study [15].

Acne, being one of the most prevalent diseases globally, has changed the stance on chronicity and complications from merely being a physiological condition to one having both pathological and psychological complications [16, 17]. Therefore, the assessment of QoL has become an important aspect of the holistic care of patients with acne. Management guidelines for acne recommended by the European Academy of Dermatology and Venereology Task Forces on Quality of Life and Patient-Oriented Outcomes recognise the use of QoL in the management of patients with acne [5]. Clinical trials on acne often use CADI as an instrument to assess QoL, and in a study conducted among acne patients referred for specialised care, it was concluded that disease-specific QoL measures are more responsive to assessing change compared to generic QoL measures [18].

The studies that used similar psychometrics for the translation and validation of the CADI questionnaire in different languages showed an internal consistency ranging from 0.7 to 0.89 [15], and are similar to the findings of this study. The highest time reported for the completion of the questionnaire in the literature was 1.5 min [15]. The time needed for the completion of CADI in Tamil doubled in our study, requiring an average of 3 min. The CADI was translated and culturally adapted for the local context, which made the questions significantly longer with additional explanations or context that are needed for the respondents to precisely understand and accurately complete the questions. The mean Tamil CADI score obtained in the study was 3.0 with a SD of 3.2. The findings of this study indicate a mild impairment in the QOL of the population studied, which is consistent with the findings where the CADI was used in non-interventional studies conducted in the community to assess quality of life [18, 19].

Regarding the dimensionality and the factor structure of the tool, among the 6 validation studies that assessed dimensionality, only those done on Nepali and Korean showed one factor [20, 21], which is comparable with the Tamil version, while the other four studies showed two factors [22–25].

Although all 5 items in the questionnaire showed a moderate to strong correlation, item 3, focusing on quality of life with truncal acne, showed the least correlation of 0.65. This, although not a significant variation, could be partly due to the lower severity of acne, the female predominance of the study sample, and the fact that it is uncommon for women to change or wear swimming costumes in public according to Sri Lankan Tamil traditions. Also, this question was pertinent for the GAGS acne severity in the chest and upper back, and 77% (*n* = 116) of the study population did not have acne on the chest and upper back.

The limitation of the study was the fact that it was implemented on a sample from the Western Province of Sri Lanka. Also, because the Tamil language is spoken in different countries, it often tends to be different on an everyday speech level due to different loanword influences and a different dialect base. Further, the presence of confounders in the study population was not evaluated, and acne, when associated with other chronic and emotional disorders, may have an impact on their QoL.

## Conclusions

The results of the present study confirm that this Tamil version of the CADI, translated and validated in Sri Lanka, shows favourable psychometric properties and should be used as a reliable and valid tool to assess QoL among Tamil language speaking patients with acne. However, achieving a truly “universal” Tamil version can be challenging due to variations in dialects, cultural nuances, and regional differences within Tamil-speaking communities. Moreover, while efforts were made to create a broadly applicable Tamil version, achieving complete universality is complex, and further research is essential to assess how well the translation performs in different contexts. As there are variations in the Tamil dialects throughout the world, it is recommended to conduct further studies on the effectiveness of this tool in assessing the QoL of patients undergoing treatment for acne in different geographical locations.

## Electronic supplementary material

Below is the link to the electronic supplementary material.

Supplementary Material 1

Supplementary Material 2

## Data Availability

The data analysis supporting the study findings are included within the manuscript. The raw datasets used during the current study are available from the corresponding author upon request.
