# Supplementary material for: Translation, cultural adaptation and validation of the Tamil version of the Cardiff Acne Disability Index (CADI) in Sri Lanka
Source: J Patient Rep Outcomes. 2024 Sep 26;8:109. doi: 10.1186/s41687-024-00782-0 (PMC11427624; doi:10.1186/s41687-024-00782-0)
Supplement: Supplementary file 1 — Supplementary Material 1 [file 41687_2024_782_MOESM1_ESM.pdf]

<u>The Cardiff Acne Disability Index (2021 Updated Version)</u>

1. As a result of having acne, during the last month have you been aggressive, frustrated or embarrassed?	☐<br>☐<br>☐<br>☐	(a) Very much indeed<br>(b) A lot<br>(c) A little<br>(d) Not at all
2. Do you think that having acne during the last month interfered with your daily social life, social events or intimate personal relationships?	☐<br>☐<br>☐<br>☐	(a) Severely, affecting all activities<br>(b) Moderately, in most activities<br>(c) Occasionally or in only some activities<br>(d) Not at all
3. During the last month have you avoided public changing facilities or wearing swimming costumes because of your acne?	☐<br>☐<br>☐<br>☐	(a) All of the time<br>(b) Most of the time<br>(c) Occasionally<br>(d) Not at all
4. How would you describe your feelings about the appearance of your skin over the last month?	☐<br>☐<br>☐<br>☐	(a) Very depressed and miserable<br>(b) Usually concerned<br>(c) Occasionally concerned<br>(d) Not bothered
5. Please indicate how bad you think your acne is now:	☐<br>☐<br>☐<br>☐	(a) The worst it could possibly be<br>(b) A major problem<br>(c) A minor problem<br>(d) Not a problem

© Cardiff Acne Disability Index. R J Motley, A Y Finlay 1992
