# Supplementary material for: Translation, cultural adaptation and validation of the Tamil version of the Cardiff Acne Disability Index (CADI) in Sri Lanka
Source: J Patient Rep Outcomes. 2024 Sep 26;8:109. doi: 10.1186/s41687-024-00782-0 (PMC11427624; doi:10.1186/s41687-024-00782-0)
Supplement: Supplementary file 2 — Supplementary Material 2 [file 41687_2024_782_MOESM2_ESM.pdf]

கார்டிவ் முகப்பரு இயலாமைக் குறியீடு (2021 புதுப்பிக்கப்பட்ட பதிப்பு)

1. முகப்பரு ஏற்பட்டதன் விளைவாக கடந்த மாதத்தில் நீங்கள் ஆக்ரோஷமாக விரக்தியாக அல்லது சங்கடமாக இருந்தீர்களா ?	அ) உண்மையில் மிகவும்<br>ஆ) நிறைய<br>இ) கொஞ்சம்<br>ஈ) இல்லவே இல்லை
2. கடந்த மாதத்தில், முகப்பரு இருப்பது உங்கள் அன்றாட சமூகவாழ்க்கை சமூக நிகழ்வுகள் அல்லது நெருக்கமான தனிப்பட்ட உறவுகளில் தலையிடுவதாக நீங்கள் நினைக்கிறீர்களா ?	அ)அனைத்து நடவடிக்கைகளையும் கடுமையாகப் பாதிக்கிறது.<br>ஆ)பெரும்பாலான செயற்பாடுகளில் மிதமான<br>இ) எப்போதவது அல்லது சில செயற்பாடுகளில் மட்டும்<br>ஈ) இல்லவே இல்லை
3. கடந்த மாதத்தில், உங்கள் முகப்பரு காரணமாக பொது மாற்றுமிட வசதிகளையோ அல்லது நீச்சல் உடைகளை அணிவதையோ தவிர்த்துள்ளீர்களா ?	அ) எல்லா நேரத்திலும்<br>ஆ) பெரும்பாலான நேரங்களில்<br>இ) எப்போதாவது<br>ஈ) இல்லவே இல்லை
4. கடந்த மாதத்தில், உங்கள் தோலின் தோற்றத்தைப் பற்றிய உங்கள் உணர்வுகளை எப்படி விவரிப்பீர்கள் ?	அ) மிக்க மனச்சோர்வும் பரிதாபகரமாகவும்<br>ஆ) பொதுவாகக் கவலையுடன்<br>இ) எப்போதாவது கவலையுடன்<br>ஈ) கவலைப்படவில்லை
5.உங்கள் முகப்பரு இப்போது எவ்வளவு மோசமாக உள்ளது என்று நீங்கள் நினைக்கிறீர்கள் என்பதைக் குறிப்பிடவும்.	அ) மிக்க மோசமான பிரச்சினையாக<br>ஆ) ஒரு பாரிய பிரச்சினையாக<br>இ) ஒரு சிறிய பிரச்சினையாக<br>ஈ) ஒரு பிரச்சினையாக இல்லை

© Cardiff Acne Disability Index. R J Motley, A Y Finlay 1992
Full details of the appropriate copyright statement are available on the
Cardiff University, Department of Dermatology website http://www.dermatology.org.uk/
